# The Importance of Leadership and Organizational Capacity in Shaping Health Workers’ Motivational Reactions to Performance-Based Financing: A Multiple Case Study in Burkina Faso

**DOI:** 10.15171/ijhpm.2018.133

**Published:** 2019-01-26

**Authors:** Amandine Fillol, Julia Lohmann, Anne-Marie Turcotte-Tremblay, Paul-André Somé, Valéry Ridde

**Affiliations:** ^1^School of Public Health, University of Montreal, Montreal, QC, Canada.; ^2^Heidelberg Institute of Global Health, Faculty of Medicine, Heidelberg University, Heidelberg, Germany.; ^3^Association Action Gouvernance Intégration Renforcement (AGIR), Ouagadougou, Burkina Faso.; ^4^IRD (French Institute For Research on sustainable Development), CEPED (IRD-Université Paris Descartes), Universités Paris Sorbonne Cités, Paris, France.; ^5^University of Montreal Public Health Research Institute (IRSPUM), Montreal, QC, Canada.

**Keywords:** Motivation, Organizational Context, Performance-Based Financing (PBF), Qualitative Research, Burkina Faso

## Abstract

**Background:** Performance-based financing (PBF) is currently tested in many low- and middle-income countries as a health system strengthening strategy. One of the main mechanisms through which PBF is assumed to effect change is by motivating health workers to improve their service delivery performance. This article aims at a better understanding of such motivational effects of PBF. In particular, the study focused on organizational context factors and health workers’ perceptions thereof as moderators of the motivational effects of PBF, which to date has been little explored.

**Methods:** We conducted a multiple case study in 2 district hospitals and 16 primary health facilities across three districts. Health facilities were purposely sampled according to pre-PBF performance levels. Within sampled facilities, 82 clinical skilled healthcare workers were in-depth interviewed one year after the start of the PBF intervention. Data were analyzed using a blended deductive and inductive process, using self-determination theory (SDT) as an analytical framework.

**Results:** Results show that the extent to which PBF contributed to positive, sustainable forms of motivation depended on the "ground upon which PBF fell," beyond health workers’ individual personalities and disposition. In particular, health workers described three aspects of the organizational context in which PBF was implemented: the extent to which existing hierarchies fostered as opposed to hindered participation and transparency; managers’ handling of the increased performance feedback inherent in PBF; and facility’s pre-PBF levels in regards to infrastructure, equipment, and human resources.

**Conclusion:** Our results underline the importance of leadership styles and pre-implementation performance levels in shaping health workers’ motivational reactions to PBF. Ancillary interventions aimed at fostering participatory as opposed to directional leadership or start-up support to low-performing health facilities will likely boost PBF effects in regards to the development of valuable motivational capacities.

## Background

Performance-based financing (PBF) aims to improve quality of and access to healthcare services by giving “*healthcare providers (facilities or health workers) financial payments based on the achievement of predetermined targets, goals or outputs after being verified for quality*” (p. 861).^1^ Usually explained in agency theory terms, PBF thereby aims to motivate healthcare providers to align their service provision behavior in the interest of the Ministry of Health, in order to maximize quality of and access to care for the population they serve.^1,2^

Although PBF has traditionally been thought to exert its motivating effect primarily through the individual financial rewards to health workers, recent research from various settings points at more complex, multifaceted, positive and negative motivational mechanisms. For instance, in Malawi, PBF motivated by increasing health workers’ perceived competence to do well in their job, by giving them goals to work towards, by increasing perceived recognition, and by changing team dynamics.^3^ In Tanzania, PBF instilled a motivating sense of competition between health workers.^4^ In Sierra Leone, PBF motivated by clarifying work tasks and objectives and by improving the working conditions.^5^ While there is a growing recognition of the importance of the implementation context in determining PBF success in the literature and applied discourse, research is yet scarce.^6-8^ In regards to health worker motivation, research points at rigid procurement channels and human resources shortages as demotivators in the context of PBF.^3,9,10^ However, there are no studies explicitly investigating how the implementation context shapes health workers’ motivational reactions to PBF yet.

This study contributes to the growing body of literature on health worker motivation in PBF by providing evidence from Burkina Faso, focusing in particular on how health workers’ perceptions of pre-existing organizational factors and PBF-induced changes therein shape health workers’ motivational reactions, using self-determination theory (SDT) as an analytical framework.^11^

### The Intervention

In Burkina Faso, PBF was initially introduced in 2011 as a pre-pilot project in 3 health districts. In December 2013, following positive evaluation,^12^ the initiative was expanded to 15 districts in six regions. The intervention is designed as a case-based payment model with a quality top-up^13,14^: Health facilities are paid subsidies according to the number of services provided from a pre-determined list of services primarily related to maternal and child health, and may receive a quality bonus if services satisfy quality norms and standards (“carrot-carrot approach”). To this end, regular verification of reported service volume and service quality is conducted in the health facilities. Health facilities are largely autonomous in deciding how to spend the additional revenue earned through PBF, with the idea being that part of it is invested in improving the facility’s infrastructure and resource situation, and part of it being disbursed to staff as bonus payments. In regards to the latter, a financial management tool is provided to ensure transparency and fairness. Individual bonuses are to be distributed across staff members based on specific criteria: qualification, seniority, responsibility, days of absence, and individual performance evaluation. Initial evidence using data from the routine health management information system indicates that the intervention had a significant positive impact on the number of postnatal consultations provided, but no impact on antenatal care consultations, completed vaccination cycles among children under the age of one, provision of modern family planning methods, and caesarian sections.^15^ A comprehensive impact evaluation based on primary data and including aspects of health worker motivation^16^ as well as aspects of quality of care is currently on-going.

Previous research on health worker motivation in Burkina Faso unrelated to the PBF program has shown high levels of intrinsic motivation and professionalism, a sense of being a health worker as a vocation, and strong feelings of duty to help the community, whereas working conditions often act as demotivators.^16,17^ Financial aspects appeared relatively unimportant as motivators pre-PBF, with salaries largely accepted especially by lower-level cadres. However, a study on health worker preferences for performance-based payment pre-implementation indicated high receptiveness of health workers for financial incentives, with the majority of respondents expressing a high preference for financial incentives overall as well as over non-financial incentives.^18^

### Conceptual Framework

SDT is increasingly used as a theoretical framework to understanding motivation in a PBF context.^19-21^ In contrast to the previously dominant intrinsic-extrinsic motivation taxonomy, SDT postulates that the level of volition leading to a certain behavior is more important than what stimulates the behavior (internal versus external stimulus).^22^ Whereas in prior conceptualizations of motivation, externally stimulated behavior was categorized as “extrinsically motivated” and contrasted with the more desirable “intrinsic motivation,” SDT posits that not the origin of the behavior stimulus is important for the quality of motivation, but rather the “perceived locus of causality” of behavior. The latter refers to a congruence of behavior with individual values, convictions, and needs. External regulations such as PBF or other interventions are often put in place to encourage individuals to engage in certain behaviors. SDT posits that so long as such externally stimulated behavior is in congruence with such individual values, convictions, and needs, a person will easily “internalize” the behavior (ie, take it on as one’s own) and experience what in SDT is called “autonomous motivation.” If behavior does not correspond to individual values, convictions, and needs, however, behavior will likely not be internalized, but rather stay “controlled motivated.” Research has consistently demonstrated autonomous motivation to be superior to controlled motivation in regards to performance as well as to a variety of other work-related outcomes such as wellbeing, organizational commitment, and turnover.^22-24^

SDT further posits that the satisfaction of 3 basic psychological needs facilitates the internalization process: (1) a sense of autonomy, ie, feeling that one is in a position to do as one thinks best at work as a health professional, within professional boundaries; (2) a sense of competence, ie, feeling that one is able to act competently and effectively at work; and (3) a sense of relatedness, ie, feeling that one is recognized, valued, and respected by patients, colleagues, and superiors.

Our study explores how PBF affects health worker motivation through its effect on the satisfaction of the basic needs for autonomy, competence, and relatedness. In particular, based on AN emerging understanding of the importance of context in population health intervention research^25^ and the wider organizational behavior literature,^26^ we postulate that pre-existing factors related to the organizational context moderate the extent to which PBF positively or negatively impacts basic needs satisfaction and thereby contribute to or rather hinder the internalization of behavior incentivized by PBF. Figure contains a graphic display of the conceptual underpinning of this article.

**Figure:**
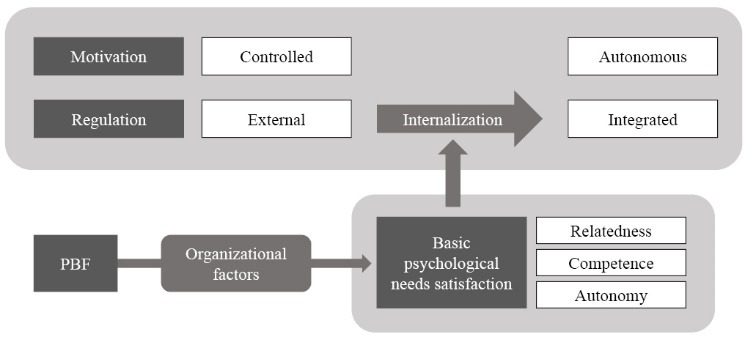
Conceptual Framework. Abbreviation: PBF, performance-based financing.

## Methods

### Research Design

The study was embedded in a larger effort to evaluate the PBF implementation process, which employed a mixed methods multiple and contrasted case study design with multiple levels of analysis, described in detail as described in Ridde et al.^14^ The study took place in 3 of the 15 PBF districts, namely Diébougou, Ouahigouya, and Solenzo, which represent well the average healthcare situation in the country. At district level, cases were selected to represent the primary and secondary levels of care, as well as different pre-PBF performance levels at primary level. In total, 2 district hospitals (Centres Médicaux avec Antennes chirurgicales; CMA) and 18 primary health facilities (Centres de Santé et de Promotion Sociale; CSPS) were sampled.

In the context of the overall process evaluation, qualitative in-depth interviews with health workers at the sampled facilities were conducted in November 2014 and February 2015, approximately one year after the start of PBF implementation in early 2014. The in-depth interviews served to capture health workers’ experiences with, reactions to, and satisfaction with the implementation of PBF in their facility.

### Sample

At primary level, all skilled clinical personnel were interviewed. At the 2 CMA, research assistants purposely selected a number of “key informants” with particularly good knowledge of the PBF implementation process in their health facility. This resulted in a total of 82 interviews, distributed across levels of care, cadres, gender, and performance (at CSPS level) as displayed in Table.

**Table:** Sample Characteristics

	**CMA**	**High-Performing CSPS**	**Low-Performing CSPS**
Level of care			
CMA	29	-	-
CSPS	-	26	27
Cadre			
Medical doctor	6	0	1
Nurse	16	15	11
Midwife/assistant midwife	6	6	9
AIS^a^	1	5	6
Gender			
Male	21	12	14
Female	8	14	13

Abbreviations: CMA, Centres Médicaux avec Antennes chirurgicales; CSPS, Centres de Santé et de Promotion Sociale. ^a^Agent itinérant de santé – mobile health worker in charge of community outreach activities.

### Data Collection Tool and Procedure

Rather than assessing motivation directly, we elicited health workers’ experiences with various aspects of the intervention (see Supplementary file 1 for an overview of interview topics). For all discussed aspects, interviewers were asked to probe respondents’ opinions and emotional reactions. We took this approach in line with previous experiences of members of the research team, who had not only found a direct and nuanced assessment of motivational changes and particularly basic needs satisfaction and autonomous motivation very difficult to implement, but also that this interview approach ‘naturally’ generated sufficient information on health workers’ motivational reactions to various aspects of PBF.^3^

Following taking of informed consent, interviews were conducted by experienced local research assistants under the coordination and supervision of a Burkinabè senior researcher with a background in socio-anthropology. All interviews were audio-recorded and then verbatim transcribed for analysis. Quotations presented below were translated into English during the manuscript writing process.

### Data Analysis

The data analysis for this article was conducted by the first author, who did not participate in the data collection process, with support from the other authors for the conceptual aspects and interpretation of results. Following an initial familiarization, the material was coded following a mix of deductive and inductive coding, along a priori codes based on the conceptual framework, the themes of the interview guide, and field notes recorded by the interview teams, but allowing additional codes to emerge while proceeding through the material. A matrix approach to coding was applied, whereby material was simultaneously coded by themes pertaining to organizational factors and by psychological needs satisfaction (relatedness, competence, autonomy). In interpreting the coded material, we triangulated responses of health workers from the same facilities with the aim of disentangling individual motivational responses from those related to the broader organizational context. The initial results were presented for discussion to 2 researchers involved in the overall process evaluation to strengthen the credibility and rigor of the analysis.^27^

## Results

### PBF: A Means to Get Health Workers to Work More and Better?

Approximately 1 year after the start of PBF, the majority of health workers expressed a sense of increased motivation and effort due to PBF and particularly the individual reward component. Most said that the anticipation of rewards made them work more or better so as to earn even more in the future. Perceived effected changes include improvements in service organization, in absenteeism, in the area of infection prevention, in documentation, and in patient reception. Many health workers further reported an increase in initiative taken by staff to correct previous substandard clinical practices. In many facilities, this included initiatives to develop clinical skills and competences in certain areas of need, which health workers appreciated not only in their current work, but also in regards to future professional development.

*“This PBF is a source of motivation, it encourages. It’s like a springboard that pushes people to give their best. […] It encourages them to make efforts even beyond their capacity. […] The more you work, the more you are recognized and the more you receive. So this encourages you to work much more in terms of quantity and also quality”* (Nurse, CMA).

Interviews revealed large differences between individual respondents in regards to their focus on the individual financial reward component as opposed to other motivating factors. For respondents with a particularly strong focus on the individual financial reward, the significant payment delays experienced several times during the implementation period as well as diverse unfairness perceptions related to reward distribution tended to overshadow any other motivational effects and acted as demotivators. For most respondents, however, negative motivational effects of the payment delays appeared to be largely contained by health workers’ professionalism and a recognition of the positive effects of PBF on their work situation.

*“We took an oath. The PBF is just a plus. For me, resources or not, it doesn’t matter much to me. The essential is that the work is done well. I’ve always told my colleagues that it’s a plus. But some people have thought from the start that PBF is a way to have a lot of money. It’s those who thought this way that [the payment delays] discourage”* (Nurse, low-performing CSPS).

Irrespective of which motivational aspects of PBF were of primary importance, virtually all health workers appeared to endorse the objectives of the intervention, which essentially reinforced adherence to pre-existing clinical protocols and guidelines, indicating a high degree of internalization.

*“Bottom line, in any case, PBF is a good thing. It allows to better take care of the patients which we receive at the CSPS. And this also allows good frequentation of the CSPS. And thirdly, it allows for us to have a small financial motivation to realize some things. There you go”* (Nurse, low-performing CSPS).

Although most respondents seem to have had largely internalized the behavior incentivized by PBF, it appears that some health workers had not fully done so at the time of the interviews approximately one year after the introduction of the intervention, as indicated for instance by the use of phrases like “… *as PBF demands …*” (Assistant midwife, high-performing CSPS) or “… *with PBF, I have no choice but to …*” (Agent itinérant de santé [AIS], high-performing CSPS).

### How Did Organizational and Management Factors Impact Basic Needs Satisfaction and Thereby Contributed to the Internalization of PBF?

Three groups of relevant organizational factors emerged during analysis, namely hierarchies, performance feedback, and resource availability.

#### Hierarchies

Many respondents spoke of the strong hierarchies in the Burkinabè health system in relation to PBF and their basic needs satisfaction, in positive as well as in negative ways. In some facilities, staff felt that the facility in-charge is the sole responsible and manager of PBF, implementing the program without involvement of or communication with other facility staff. Many health workers knew of the program, but could not provide any details on its design and implementation. This was particularly problematic in regards to the individual financial rewards (locally referred to as subsidies) among staff members, which staff from some facilities felt was a completely untransparent process.

*“For [the subsidies], I really don’t know. Because, you see, our in-charges, it’s them who manage. And we are only waiting for the day when they come and tell us our subsidies. If all us health workers could be involved, it would make us happy. But we do not know. PBF, it is true that we have subsidies, but people here do not know the timing of payment of these subsidies: is it monthly, every 2 months, we do not know. It’s the in-charge who holds these secrets”* (Assistant midwife, high-performing CSPS).

For some respondents, this reinforced pre-existing feelings of powerlessness regarding their influence on facility matters and their valorization as valuable and knowledgeable facility members, as expressed by phrases like *“anyway, we’ll never be listened to”* (Assistant midwife, low-performing CSPS) and “*they are our employers, what can we do?*” (Midwife, low-performing CSPS), negatively impacting the satisfaction of their basic need for autonomy and relatedness.

In other facilities, in contrast, good pre-existing team work and comparatively flat hierarchies supported a participatory and transparent introduction of PBF which in turn supported health workers’ basic needs satisfaction. In one high-performing CSPS, for instance, one nurse spoke with enthusiasm about the “*participative spirit of the PBF, we do service meetings, we explain, everyone knows how to dance now*.” In the same facility, another nurse expressed his appreciation of this new participative spirit by speaking about how “*as soon as the money is transferred, we sit in a meeting, we calculate together, we do the different parts, absences, holidays and everything, we subtract and according to the qualifications, each of us knows what to take. Really, here, we do everything together,*” signaling positive effects on the satisfaction of his need for relatedness. The facility’s assistant midwife described how “*there were things we did not do, at first it was difficult anyway [...] but it became water to drink. Like that, really, if you now see a woman, you manage to do that yourself, you’re proud of yourself, you know you took care of her,*” signaling a positive contribution to the satisfaction of her need for competence.

At least in one case, PBF appeared to have provided an opportunity to break previous strong hierarchies by moving health workers’ focus away from hierarchies and “formal say” to facility performance. A lower-level cadre health worker reported that contrary to previous behavioral norms, with PBF, he felt comfortable advising his superior on facility management issues, contributing positively to his basic needs for autonomy and relatedness.

“* When RBF came, I told the major: ‘You should diminish absenteeism, otherwise, the maternity will become worse rather than better’” (AIS, high-performing CSPS).*

Rigid hierarchies appeared to impact basic needs satisfaction not only in regards to within-facility relationships, but also in regards to the intervention more generally. Many health workers felt that PBF was introduced by directive and without adequate regard for their work realities in many ways, negatively contributing to their basic needs for relatedness and competence.

*“Now, there is a lot of documentation. If you do not, when the verifier comes, it’s as if you did not do anything, it’s 0. So you have to take time and when you take time, there are patients who complain, […] the more people are waiting outside. When I was in the East, there was no PBF. From 07:00 to 14:00, we took no less than 100 patients, but we cannot do it here. If you do, you will not be paid”* (In-charge, low-performing CSPS).

*“PBF does not buy all services. For those whose services are not bought, it’s like their work is not valued […]. It’s a frustration”* (Nurse, CMA).

A particular case was the AIS, who in principle are not supposed to provide curative care or perform deliveries and do not learn how to do so in their initial training, but who in practice often perform these tasks in light of the general staff shortage and who have often received respective in-service training.

*“When PBF came, they said that deliveries were done by an unqualified health worker: we are treated as unskilled health workers. And yet, an AIS can properly deliver, better than an assistant midwife with 2 years of service. I have 7 years of service in the maternity ward, a midwife who has just been out [of school] cannot do what I am doing. She is more than me, but what I know in maternity, she does not know!”* (AIS, high-performing CSPS).

#### Performance Feedback

Although formal performance feedback processes had been in place even before the introduction of PBF, PBF acted to reinforce their regular implementation which had been an issue before. Some health workers described appreciating both positive and negative feedback, viewing it as recognition of their work and constructive advice on how to improve even further, strengthening the satisfaction of their need for competence and relatedness.

*“What I like in PBF is not the resources we receive. Anybody who does a job and then someone comes to tell you, “your work, it’s impeccable,” it’s an unmeasurable satisfaction. But if someone comes to tell you that what you’re doing there, it’s not good, it’s a total disappointment. So for me personally, PBF has led us to work in quantity and quality. [The feedback], it is a satisfaction for us, it is a source of motivation”* (AIS, high-performing CSPS).

Some health workers, in contrast, expressed having felt incompetent and even ashamed in response to negative feedback. For instance, one health worker described how negative evaluations had affected his perceived competence.

*“On audit day, when we get zeros, we look at each other […] it’s shameful. We’re all there, we attend, we say that over here and over there, it’s not working, so zero, zero, and you’re traumatized. It’s really mortifying for you”* (AIS, low-performing CSPS).

The extent to which feedback was constructively received and contributed to basic needs satisfaction appeared to depend much on the facility in-charges leadership and supervisory style. For example, in one facility, a health worker explained how the in-charge actively involved him, describing how this made him feel recognized and empowered, strengthening his need for competence and relatedness.

*“One wants to agree with one’s evaluation. That it is clear what you did well, what you did wrong, why you deserve the evaluation you received. There should be consensus. To me, it’s a responsibility”* (Nurse, CMA).

#### Resource Availability

Experiences in regards to material resources were mixed across facilities. Whereas it appears that some facilities used the PBF funds for improvements in the availability of medico-technical supplies, positively impacting health workers’ perceived ability to work well and thereby strengthening their basic need for competence, this seems to have not been the case in others.

*“Health workers now have good working conditions: clean work places, material available”* (Nurse, high-performing CSPS).

*“The difficulties we encounter in the implementation of PBF, at least in my maternity, is that material is lacking. We did everything we could to have them, but there is always something missing. They say we should have at least 4 or 5 delivery beds, but this is complicated for us”* (Assistant midwife, low-performing CSPS).

One problem common to all health facilities and persisting with PBF was a perceived shortage of human resources. In some health facilities, health workers explicitly spoke about how they could not achieve PBF objectives due to lack of staff. PBF had created this sense of increased workload by making certain technical resources available and enforcing close adherence to treatment guidelines, thereby increasing time spent on consultations including documentation (eg, partographs, Integrated Management of Childhood Illness [IMCI] checklist). Many health workers were ambivalent in their reactions to this new situation, appreciating and endorsing the intervention aims as indicated above, but simultaneously perceiving the increased workload as overburdening and hindering adequate fulfillment of all tasks, weighing on their need for competence.

*“We do not have the personnel required for [work of high quality]. If you are in the dispensary and you have to be there to consult from 8am to 5pm, and in the same time the midwife is alone with more than 60 children for growth monitoring. When there is not too much to do at the dispensary, we can help, because […] there really is a lot to do. […] Most CSPS are made up of a nurse, an AIS, and a midwife. It goes without saying that when the nurse is not there, the quality of consultation is not there. The staff is so small that very often, when they come for quality evaluation, it makes us lose a lot of points”* (Nurse, high-performing CSPS).

## Discussion

Using SDT and the concept of basic psychological needs, our study aimed to shed light onto how PBF as implemented into pre-existing work realities can promote or hinder the internalization of behavior incentivized by PBF, fostering or thwarting positive autonomous motivation.

As hypothesized, results show that the extent to which PBF positively impacted motivation depended to quite some extent on the “ground upon which PBF fell,” ie, on health workers’ experiences of their context, beyond health workers’ individual personalities and disposition. Two aspects emerged as particularly relevant in health workers’ perception, namely the facility managers’ leadership behavior and the facility’s pre-implementation resource situation.

### Key Importance of Leadership

Health workers’ perceptions of their facility managers’ leadership skills and style appeared to be of high importance for a constructive PBF implementation process and subsequent positive effects on health workers’ basic needs satisfaction, similarly to what has been found in relation to other prior interventions in Burkina Faso as well as in the broader literature on work motivation.^28-30^ For the case of Burkina Faso and similar contexts with a strong presence of hierarchical leadership styles, our results imply that leadership support and training in the context of PBF or similar health system interventions might be highly conducive to the development of positive and lasting forms of work motivation. By helping leaders in the transition from directive to participatory leadership styles, PBF program implementers can support building of feelings of valorization, trust and perceived competence, thereby ultimately propelling performance in the context of PBF and beyond.^30-31^ Simple strategies might include encouraging staff members to voice their opinions and ideas in a judgement- and consequences-free environment and making facility-related decision-making transparent, for instance in the context of regular staff or supervisor-subordinate meetings.

### PBF: Success (Only) to the Successful?

One key assumption of PBF is that performance-contingent provision of resources will allow health facilities to gradually improve their performance in bottom-up fashion.^32^ In practice, it has long been recognized that a certain baseline performance level particularly in terms of infrastructure and large-scale resources might be required for PBF to trigger positive cycles or improvement.^32^ Renmans et al speak of “success to the successful” in a recent publication.^33^ Many PBF interventions have therefore entailed an initial start-up component to bring facilities up to a certain performance level from which they would then be able to further transform themselves.^32^ In Burkina Faso, such an initial start-up component had originally been planned,^13^ but was later not implemented due to budgetary constraints.

Our results align with these experiences in that in tendency, facilities with sufficient pre-PBF capacity particularly in terms of human resources and leadership seemed relatively successful in developing their performance with PBF in the eyes of the respondents, with largely positive consequences for basic psychological needs satisfaction. Facilities with weak pre-PBF capacity, in contrast, appeared stuck and unable to “dig themselves out” of their low-performing situation. This was not only not conducive to positive forms of motivation, but rather thwarted health workers’ basic needs for competence, relatedness, and autonomy.

### Limitations of the Study

Our study has several important limitations. First, it relies on a case-study approach, with facilities having been selected in the context of the broader process evaluation study and based on their pre-implementation performance, notably in terms of average numbers of patients per month. Many organizational factors, which are at the center of our analysis, could unfortunately not be taken into account, although we did observe sufficient variation in health workers’ description to be reasonably confident that various pre-PBF contexts are adequately represented in the sample. We did not measure organizational factors objectively in the context of the data collection. Rather, the study describes health workers’ perceptions of organizational factors and their role in shaping their motivational reactions to PBF. Although there was often a high degree of congruence in perceptions among staff from the same health facility, in interpreting the findings, it is important to recall that perceptions do not necessarily reflect the objective situation. Second, as motivation was only one of several subjects and objectives of the in-depth interviews, there was limited time to understand motivational impacts. None of the respondents spoke explicitly about impact on their basic needs satisfaction – which was expected from prior experiences^3^–, so that we had to resort to inferring from reported experiences and behavior, which poses certain risks of misinterpretation. Third, analysis was led by a researcher not directly involved in data collection and with an important time lag. Although other authors were involved in data collection and could provide field contextualization, this precluded using an iterative approach which might have allowed more depth in the exploration of motivational mechanisms and effects. Fourth, data was collected one year after the start of the intervention, when the intervention was likely still in the process of maturing and motivational effects were likely still to change over time. Finally, it is likely that in addition to other individual factors, motivational reactions of health workers differ much according to their pre-intervention motivation levels. Unfortunately, we did not have such information available for a respective additional analysis.

## Conclusion

While the crucial importance of the implementation context is increasingly recognized in the PBF community, explicit research is yet scarce, especially on contextual factors as moderators of the motivational effects of PBF. Our study contributes to filling this gap in knowledge by providing evidence on the importance of perceived leadership styles and pre-implementation performance levels in shaping health workers’ motivational reactions to PBF. Ancillary interventions aimed at fostering participatory as opposed to directional leadership or start-up support to low-performing health facilities might therefore not only boost PBF effects in regards to quality of care, but also foster the development of positive, sustainable forms of autonomous motivation.

## Acknowledgements

The research project is part of the “Community research studies and interventions for health equity in Burkina Faso.” We thank the Canadian Institutes of Health Research (CIHR) for funding the program (Grant number ROH-115213). VR holds a CIHR-funded Research Chair in Applied Public Health. AMTT obtained a bursary from the University of Montreal Hospital Research Center and the University of Montreal Public Health Research Institute. AMTT received a training bursary from the CIHR. This study would not have been successful without the contributions of Maurice Yaogo and Sylvie Zongo, who managed and supervised the data production, and Guillaume Kambire, Saliou Sanogo, Mariétou Ouedraogo, Souleymane Ouedraogo, Vincent Paul Sanon, Idriss Gali Gali, Vincent Koudougou, Assita Keita, Maimouna Sanou, Elodie Ilboudo, and Ahdramane Sow, who collected data in the health centers. We also wish to thank Donna Riley, who translated a previous version of the manuscript.

## Ethical issues

Ethical approval was obtained by the health research ethics committees of Burkina Faso (February 14, 2014) and the University of Montreal Hospital Research Centre (CRCHUM) (13.158). The Ministry of Health approved and endorsed the study.

## Competing interests

JL is a researcher on the impact evaluation of PBF in Burkina Faso. Her salary as an employee at Heidelberg University is funded through a research grant of the PBF intervention funder (World Bank). VR is a co-researcher on the baseline impact evaluation but receives no salary from the World Bank. None of the authors received any payment from the World Bank for the presented analysis or writing of the manuscript. The World Bank did not interfere in study design, data analysis, or writing in any way.

## Authors’ contributions

The overall process evaluation in which this study is embedded was conceived by VR, PAS, AMTT, and JL. PAS and AMTT supervised the data collection. AF and VR conceived the analytical strategy. The analysis was carried out by AF, with support from JL. All authors provided input into the final interpretation of results. AF and JL drafted the manuscript. All authors provided input into and approved the final manuscript.

## Authors’ affiliations

^1^School of Public Health, University of Montreal, Montreal, QC, Canada. ^2^Heidelberg Institute of Global Health, Faculty of Medicine, Heidelberg University, Heidelberg, Germany. ^3^Association Action Gouvernance Intégration Renforcement (AGIR), Ouagadougou, Burkina Faso. ^4^IRD (French Institute For Research on sustainable Development), CEPED (IRD-Université Paris Descartes), Universités Paris Sorbonne Cités, Paris, France. ^5^University of Montreal Public Health Research Institute (IRSPUM), Montreal, QC, Canada.

## Supplementary files


Supplementary files 1. Interview topics in-depth interviews with health workers.

## Key messages

**Implications for policy makers**

- Organizational context factors moderate health workers’ motivational reactions to performance-based financing (PBF).
- Ancillary interventions aimed at fostering participatory leadership or start-up support to low-performing health facilities have the potential to positively boost health workers’ motivational reactions to PBF.

**Implications for public**

Performance-based financing (PBF) aims at improving the quality of and access to healthcare services by motivating health workers to improve their performance. In practice, various factors often hinder intervention success. The study investigated how the organizational context into which PBF was implemented in Burkina Faso shaped health workers’ motivational reactions. Our findings suggest that additional elements such as leadership training might boost PBF’s potential to foster health worker motivation, and therefore positively contribute to patients’ healthcare.
