# Supplementary material for: The Importance of Leadership and Organizational Capacity in Shaping Health Workers’ Motivational Reactions to Performance-Based Financing: A Multiple Case Study in Burkina Faso
Source: Int J Health Policy Manag. 2019 Jan 26;8(5):272–9. doi: 10.15171/ijhpm.2018.133 (PMC6571493; doi:10.15171/ijhpm.2018.133)
Supplement: Supplementary file 1 — Supplementary files 1. Interview topics in-depth interviews with health workers. [file ijhpm-8-272-s001.pdf]

Supplementary File 1. Interview topics in-depth interviews with health workers

1. Respondent characteristics
    a. Unguided self-presentation
    b. Cadre
    c. Responsibility level
    d. Duty station
    e. Number of years in service
    f. Implication in PBF
2. Work organization and functioning of the health facility
    a. Service organization / distribution of tasks
    b. Number of health workers present
    c. Exact work tasks
    d. Number of patients per day
    e. Most frequent causes for consultations
3. PBF
    a. General knowledge about PBF
        i. Origin
        ii. Objective
        iii. Functioning
        iv. Involved actors
        v. Activities
        vi. Performance indicators
        vii. Performance contracts
        viii. Incentives
        ix. Information sources
    b. Training in PBF
        i. Location
        ii. Period
        iii. Trainers
        iv. Content (if retained)
        v. Frequency
        vi. Perceptions
    c. Local emergence and implementation of PBF

i. Start of the intervention
ii. Actors
iii. Description of the implementation process
iv. Activities
v. Identification of performance indicators
vi. Performance contracts
vii. Modalities of incentive calculation
viii. Modalities of incentive payment
ix. Role of the community
x. Terminology used
xi. Adaptation strategies
xii. Contextual facilitators/barriers
xiii. Reactions of the different actors (health workers, community)

d. Perceptions of PBF
   i. Utility, interest
   ii. Organization of daily work tasks
   iii. Accessibility and quality of health care services
   iv. Frequentation of health facilities
   v. Competition between staff members/health facilities
   vi. Motivation of staff (absenteeism, effort)
   vii. Practices of care
   viii. Working conditions
   ix. Workload
   x. Community participation
   xi. Unexpected situations

e. Difficulties encountered in the implementation of PBF
   i. Implication of health workers
   ii. Implications of the community
   iii. Availability of resources
   iv. Definition of indicators
   v. Establishing performance contracts
   vi. Payment of performance incentives vs salaries
   vii. Accessibility of services and medication
   viii. Management of PBF and non-PBF activities

ix. Supervision/monitoring of activities
x. Adherence to timelines

(the following pertained to ancillary components of PBF and was not used for this analysis as no direct implications for motivation were apparent upon screening the material)

f. Targeting and user fee exemption of the ultra-poor
    i. Identification
    ii. Implementation
    iii. Training
    iv. Community reactions
    v. Health worker reactions
    vi. Opinions
g. Community-based health insurance
    i. Knowledge
    ii. Training
    iii. Community reactions
    iv. Health worker reactions
    v. Opinions
h. Interaction of PBF with targeting of the ultra-poor and community-based health insurance
    i. Targeting process
    ii. Implication therein
    iii. Community reactions
    iv. Opinions
    v. Difficulties encountered
